# Probiotic Potential of *Clostridium* spp.—Advantages and Doubts

**DOI:** 10.3390/cimb44070215

**Published:** 2022-07-07

**Authors:** Tomasz Grenda, Anna Grenda, Piotr Domaradzki, Paweł Krawczyk, Krzysztof Kwiatek

**Affiliations:** 1 Department of Hygiene of Animal Feeding Stuffs, National Veterinary Research Institute, Partyzantow 57, 24-100 Pulawy, Poland; kwiatekk@piwet.pulawy.pl; 2 Department of Pneumonology, Oncology and Allergology, Medical University in Lublin, Jaczewskiego 8, 20-950 Lublin, Poland; anna.grenda@umlub.pl (A.G.); pawel.krawczyk@umlub.pl (P.K.); 3 Department of Commodity Science and Animal Raw Materials Processing, University of Life Sciences in Lublin, Akademicka 13, 20-950 Lublin, Poland; piotr.domaradzki@up.lublin.pl

**Keywords:** *Clostridium*, commensal Clostridia, probiotic, *Clostridium* clusters

## Abstract

*Clostridium* spp. is a large genus of obligate anaerobes and is an extremely heterogeneous group of bacteria that can be classified into 19 clusters. Genetic analyses based on the next-generation sequencing of 16S rRNA genes and metagenome analyses conducted on human feces, mucosal biopsies, and luminal content have shown that the three main groups of strict extremophile anaerobes present in the intestines are *Clostridium* cluster IV (also known as the *Clostridium leptum* group), *Clostridium* cluster XIVa (also known as the *Clostridium coccoides* group) and *Bacteroides*. In addition to the mentioned clusters, some *C. butyricum* strains are also considered beneficial for human health. Moreover, this bacterium has been widely used as a probiotic in Asia (particularly in Japan, Korea, and China). The mentioned commensal Clostridia are involved in the regulation and maintenance of all intestinal functions. In the literature, the development processes of new therapies are described based on commensal Clostridia activity. In addition, some Clostridia are associated with pathogenic processes. Some *C. butyricum* strains detected in stool samples are involved in botulism cases and have also been implicated in severe diseases such as infant botulism and necrotizing enterocolitis in preterm neonates. The aim of this study is to review reports on the possibility of using *Clostridium* strains as probiotics, consider their positive impact on human health, and identify the risks associated with the expression of their pathogenic properties.

## 1. Introduction

The genus *Clostridium* is comprised of anaerobic, Gram-positive, rod-shaped, spore-forming bacteria. The first isolation of *Clostridium* spp. was described in the nineteenth century by Louis Pasteur. He noticed that the new bacterium was able to grow under anaerobic conditions. This microorganism was named *Vibrion butyrique* because of its ability to produce butyric acid via anaerobic butyric fermentation. At the end of the 19th century, Adam Prażmowski renamed the discovered microorganism *Clostridium butyricum* [1]. Clostridia are ubiquitous in the environment, where they mainly inhabit soil and water sediment, but also the alimentary tract of humans and animals. The bacteria of the genus *Clostridium* are often described only as a biological threat and an enemy of mankind. Many of them, however, have positive properties and they can be used in many industries, and also to support different kinds of therapies that are beneficial for human and animal health [1,2]. Clostridia perform a variety of metabolic functions, including the conversion of starch, proteins, and purines into organic acids (i.e., acetic, butyric, and caproic acid), alcohols, CO_2_, and hydrogen. *Clostridium* spp., due to their broad and flexible metabolic capacity, are part of many ecosystems of microbial co-culture [3]. These bacteria include groups of very heterogeneous microorganisms. Based on 16S rRNA analyses, a new taxonomic criterion based on phylogenetic analyses has been presented. A total of 19 clusters or groups were identified, leading to the description of five new genera and the proposition of eleven new species combinations [4]. Subsequent studies confirmed and extended this structure, with many organisms formally recognized as species of the genus *Clostridium* being transferred to new or established genera [5]. The new criterion introduced some asporulate bacteria, such as *Roseburia cecicola* and *Ruminococcus torques* (25). Most previous members of *Clostridium* were assigned to *Clostridium* cluster I, represented by *C. butyricum*. The reclassification of organisms from the genus Clostridium still continues, and all changes are updated on a regular basis on the List of Prokaryotic Names with Standing in Nomenclature (http://www.bacterio.net, accessed on 5 May 2022) [5].

The human and animal alimentary tract is inhabited by about 1 kg of commensal microbes. These commensals belong to *Bacteria*, *Archea*, *Eucarya*, and viral particles. Recent advances in next-generation sequencing (NGS) have made it possible to perform phylogenetic 16S rRNA and metagenome analyses of samples from mucosal biopsies, luminal content, and human feces, revealing that 98% of intestinal microbiota are comprised of four major bacterial phyla: *Firmicutes*, *Bacteroides*, *Protobacteria* and *Actinobacteria* [6,7]. Among these phyla, three main groups of extremophile anaerobes are recognized: *Bacteroides*, *Clostridium* cluster XIVa (*Clostridium coccoides* group), and *Clostridium* cluster IV (*Clostridium leptum* group) [8,9,10,11,12,13,14]. The mentioned groups of *Clostridium* spp. make up a huge share of the total intestinal microbiota (estimated at 10–40%) [6,10,15,16]. In addition to the mentioned clusters, some *C. butyricum* strains (belonging to cluster I) are also considered to be beneficial for human health, and they represent approximately 10–20% of human fecal samples, as revealed by microbial culture. Moreover, this bacterium has been widely used as a probiotic in Asia (particularly in Japan, Korea, and China). Clostridia are able to produce many beneficial substances for human and animal health, such as organic acids (acetic, butyric, fumaric, and lactic) [1]. Aside from the benefits linked to the positive impact of *Clostridium* on intestinal homeostasis, this genus still creates doubt because of its possible involvement in the formation of dangerous toxins and its pathogenic activity toward humans and animals. *Clostridium* spp. are still seen in a positive light as members of the probiotic family.

The aim of this study is to present the potential health benefits of clostridial activity (especially *C. leptum*, *C. coccoides* groups, and *C. butyricum*), and also the risks carried by the expression of their pathogenic properties.

## 2. *Clostridium leptum* and *Clostridium coccoides* Groups—Beneficial Activity

**Figure 1:**
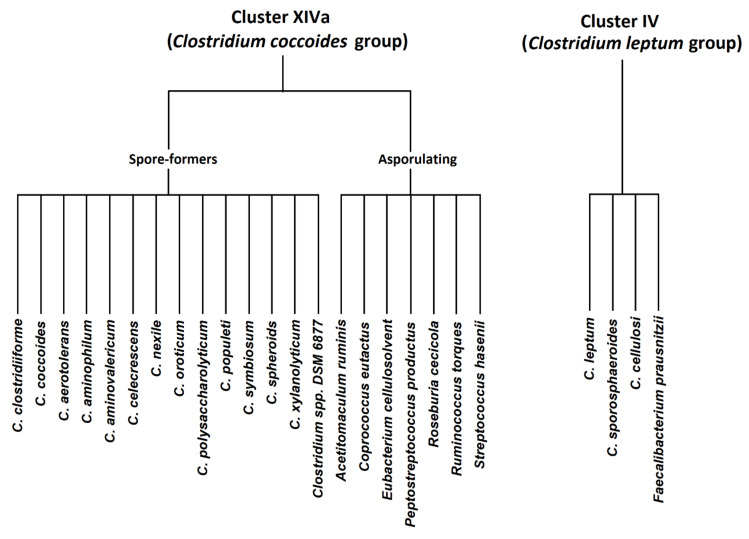
*Clostridium coccoides* and *Clostridium leptum* groups—graphical presentation.

Members in cluster IV (*C. leptum* group) include *C. leptum*, *C. sporosphaeroides*, *C. cellulosi*, and *Faecalibacterium prausnitzii*. *Clostridium* cluster XIVa (*Clostridium coccoides* group) consists of 21 species, including spore-forming Clostridia (*C. coccoides*, *C. aerotolerans*, *C. aminophilum*, *C. aminovalericum*, *C. celecrescens*, *C. nexile*, *C. oroticum*, *C. polysaccharolyticum*, *C. populeti*, *C. symbiosum*, *C. spheroids*, *C. xylanolyticum*, and *Clostridium* spp. DSM 6877) and other asporulate species (*Acetitomaculum ruminis*, *Coprococcus eutactus*, *Eubacterium cellulosolvens*, *Peptostreptococcus productus*, *Roseburia cecicola*, *Ruminococcus torques* and *Streptococcus hansenii*) [2]. The members of both groups are presented graphically in Figure 1.

*Clostridium coccoides* is considered to be one of the most predominant groups of bacteria in the human intestines. This group consists of different species such as *Clostridium*, *Butyrivibrio*, *Dorea*, *Coprococcus*, *Eubacterium*, *Ruminococcus* and *Roseburia*, which are classified as high oxygen-sensitive anaerobes. Some of these bacteria are known to be butyrate-producing. The *Clostridium coccoides* group (cluster XIVa) constitutes almost 60% of mucin-adhered microbiota. The Cluster XIVa group is shown to prevent vancomycin-resistant *Enterococcus* (VRE) colonization, as demonstrated in an antibiotic-treated mouse model [17,18]. The mentioned groups of Clostridia and their beneficial effects are listed in Table 1.

Cluster IV (*Clostridium leptum* group) is considered to be dominant in adult human fecal microflora—its abundance approaches about 16–25%. Characteristic for this group is a synergy with different intestinal microbes in unabsorbed dietary carbohydrate fermentation. The production of short-chain fatty acids (SCFAs) by these bacteria is the major source of energy for the colonic epithelium, consequently regulating intestinal epithelial function. *Faecalibacterium prausnitzii* is considered to be the most frequently encountered and abundant member of the *C. leptum* group. A significant production of butyrate through carbohydrate fermentation is attributed to this species. This bacterium is also considered to play an anti-inflammatory function in the gastrointestinal tract. An imbalance in the composition of intestinal microbiota results in the potential for the development of serious gastrointestinal diseases, such as inflammatory bowel diseases (IBD). Ulcerative colitis and Crohn’s disease (CD), in particular, are suspected to result from an imbalance in the natural immune reaction to the luminal microbiota in some individuals, along with genetic disorders [19,20,21,22,23,24]. The mentioned alterations are frequently associated with a significant increase in *Bacteroides-Prevotella*, an increase or decrease in bifidobacterial, and a decrease in the *C. leptum* group, especially *F. prausnitzii*. The mentioned changes were noticed in patients with active Crohn’s disease [25]. *Faecalibacterium prausnitzii* was also proven to prevent inflammation by blocking the nuclear factor kappa-light-chain-enhancer of activated B cells (NF-κB) and the release of interleukin 8 (IL8). This bacterium was also recognized for its anti-inflammatory effect caused by chronic colitis [26].

Umesaki et al. [27] and Atarashi et al. [28] observed that a mixture of commensal Clostridia was able to change the intraepithelial lymphocyte profile in the large intestine. Atarashi et al. [29] noticed that *C. leptum* and *C. coccoides* groups were able to induce the accumulation of mucosal Treg cells in the colon and enrich transformation growth factor-β in the colon. They also noticed that the chosen strains of IV, XIVa and XVIII clusters could induce the expansion and differentiation of regulatory Treg cells. The authors reported that the oral administration of a mixture of the mentioned clusters could attenuate allergic diarrhea and colitis in mice [29]. A study by Godefroy et al. [30] suggested that DP8α cells (among them, DP8α T cells, including *F. prausnitzii*-specific T cells have been recognized) co-expressing CCR6 and CXCR6 cause a decrease in inflammation in inflammatory bowel disease (IBD) patients. *Faecalibacterium prausnitzii* is able to release an extracellular polymeric matrix (EPM) and can form a biofilm. The EPM is able to trigger the secretion of IL-10 and IL-12, which attenuate inflammation [31]. Moreover, salicylic acid could be produced from salicin fermentation by 40% of *F. prausnitzii* strains and block the production of IL-8 [32].

In recent decades, a lot of data have appeared on the 16S rRNA-based profiling of gut microbiota connected to the abundance of the *C. leptum* group, although the most data are connected to *F. prausnitzii* and its health benefits in IBD (Crohn’s disease, ulcerative colitis) [20,21,24,33,34,35]. *Faenicalibacterium prausnitzii* is considered to be an indicative factor (biomarker) of the intestinal condition of the adult human body, and has also been recognized as a prospective predictive factor in Crohn’s disease (CD). An *F. prausnitzii* deficiency was observed in patients with Crohn’s disease in the ileum [23]. It was noticed that a low level of *F. prausnitzii* in ileal CD patients undergoing surgery was associated with a higher risk of postoperative recurrence [23,36]. Swidsinski et al. [24] invented a diagnostic test based on the detection of *F. prausnitzii* and leukocyte count. This test made it possible to distinguish active CD from ulcerative colitis (UC) with sensitivity at the level of 79–80% and a specificity of 98–100%. In the UC patients, a significant increase in leukocytes together with high *F. prausnitzii* numbers was observed [24]. Bacterial dysbiosis correlated with IBD, with a visible imbalance of the mucosal protective bacteria predominantly in the *C. leptum* group, including *F. prausnitzii* [23]. Macafferi et al. [34] reported on the application of rifaximin to CD patients causing remission, and noticed an increase in the level of bifidobacteria and *F. prausnitzii*. Dorffel et al. [15] showed that other types of treatment such as chemotherapy and interferon a-2b caused the depletion of *F. prausnitzii*. Additionally, the high-dose cortisol therapy of infliximab is able to restore the number of *F. prausnitzii* from an undetectable level up to 1.4 × 10^10^ bacteria/mL within several days [24,37]. The mechanism for modulating the number of *F. prausnitzii* in patients undergoing treatment for IBD is still unexplained.

**Table 1:** Cluster IV (*Clostridium leptum* group) and Cluster XIVa (*Clostridium coccoides* group) probiotic properties.

Cluster		Species	Beneficial Properties	Literature
Cluster IV (*Clostridium leptum* group)		*C. leptum* *C. sporosphaeroides* *C. cellulosi* *Faecalibacterium prausnitzii*	Production of SCFAs, especially butyrate; Regulates intestinal epithelial function; Performs an anti-inflammatory role in the gastrointestinal tract (blocking NF-κB and IL-8 production); Induces Treg cell accumulation in the colon; Attenuates allergic diarrhea and colitis; Triggers the secretion of IL-10 and IL-12 which attenuate inflammation.	[2,7,8,9,10,11,14,15,19,20,21,22,23,26,27,30,31,32]
Cluster XIVa (*Clostridium coccoides* group)	Spore-formers	*C. clostridiiforme* *C. coccoides* *C. aerotolerans* *C. aminophilum* *C. aminovalericum* *C. celecrescens* *C. nexile* *C. oroticum* *C. polysaccharolyticum* *C. populeti* *C. symbiosum* *C. spheroids* *C. xylanolyticum* *Clostridium* spp. DSM 6877	Produces SCFAs; Prevents vancomycin-resistant *Enterococcus* (VRE) colonization; Performs an anti-inflammatory role in the gastrointestinal tract (blocking NF-κB and IL-8 production); Induces Treg cell accumulation in the colon.	[2,8,9,10,11,13,14,17,18]
	Asporulating	*Acetitomaculum ruminis* *Coprococcus eutactus* *Eubacterium cellulosolvent* *Peptostreptococcus productus* *Roseburia cecicola* *Ruminococcus torques* *Streptococcus hasenii*		

## 3. *Clostridium butyricum*—Its Probiotic Properties and Ability to Support Treatment

Aside from the groups of Clostridia mentioned above, some strains of *C. butyricum* (belonging to cluster I) are considered to be probiotic as well. *C. butyricum* strains are found in various niches, including soil, vegetables and fermented dairy products, and are also natural inhabitants of the intestines of humans and animals. *Clostridium butyricum* is able to produce SCFAs by fermenting undigested dietary fiber, especially butyrate and acetate. Butyrate is considered to be one of the main fermentation products released by *C. butyricum* in the butyrate kinase (buk) pathway [38,39]. Short-chain fatty acids released in the colon by microorganisms exert a myriad of effects on host health, including modulating immune homeostasis in the gut, enhancing gastrointestinal barrier function, and alleviating inflammation. *Clostridium butyricum* has been isolated from 10–20% of the mature human population and is also considered one of the primary colonizers found in the intestines of infants. It has been shown that *C. butyricum* is able to survive the high acidity of the gastrointestinal environment. Literature data indicate the beneficial effects of the application of *C. butyricum*, such as promoting faster animal growth and enhancing different immune functions as well as microecological balance [38,39,40]. A preventive effect against *Esherichia coli* and *Clostridioides difficile* infections and its influence on the reduction in intestinal damage and permeability were demonstrated [41]. 

Some non-pathogenic strains of this species have been used as probiotics for decades, e.g., *C. butyricum* MIYAIRI 588 isolated from human stool samples by Chikaji Miyairi in 1933 and in 1963 from soil samples [42]. This strain is a probiotic commercially available in Japan and Korea, and is used for supporting the treatment of antimicrobial-associated diarrhea [42]. Moreover, the mentioned strain was also authorized as a novel food ingredient by the European Parliament and the Council [43]. *Clostridium butyricum* seems to be a promising candidate in curing dysbiosis caused by various diseases, such as gastrointestinal, neurological and metabolic disorders, and cancer. The mechanism of its beneficial properties is not fully elucidated; however, some evidence has appeared in the literature linking benefits with the production of SCFAs and their pathways to impact immune homeostasis, as well as the physiology of the gut barrier. It is also possible that *C. butyricum* has an influence on increasing the number of probiotic bacterial taxa, such as *Lactobacillus* and *Bifidobacterium* [39,40,42,44,45].

The intestinal barrier plays an important role in maintaining intestinal microbiota tolerance, nutrients, electrolytes, and water absorption, and maintaining the required level of defense against pathogen invasion. It is a specific selective gate that prevents the diffusion of toxins or antigens. The intestinal barrier consists of three layers: the mucus layer, epithelium, and lamina propria [32,46]. The mucus is considered to be the first physical barrier against pathogens. This layer varies in thickness in the different parts of the gastrointestinal tract, and is mainly composed of glycoproteins such as mucins which are secreted by the epithelium [33]. The disintegration of mucin in mice is conductive to the development of colitis and increases the probability of the development of colorectal cancer [47,48]. The protective abilities of *C. butyricum* have been tested in two mouse model experiments. Long et al. [49] described a significant increase in colonic mucosal thickness after *C. butyricum* Sx-01 administration. Hagihara et al. [39] also noticed that the application of *C. butyricum* MYRIAYRI 588 caused an increase in mucin secretion and significantly decreased epithelial damage in colonic tissues during recovery from clindamycin-induced antibiotic-associated diarrhea. The abundant production of butyrate by *C. butyricum* strains is indicated as the main factor for the increase in mucin secretion, which results in the increased expression of the mucin MUC genes. *Clostridium butyricum* also has an influence on TJ protein (molecules situated at the tight junctions of the epithelial, endothelial, and myelinated cells) expression across several different disease models [45,50]. The positive effect of *C. butyricum* on the intestinal epithelium is attributed to butyrate production. The fecal concentration of butyrate increases significantly after *C. butyricum* supplementation. Additionally, direct supplementation with sodium butyrate showed a beneficial effect on increased intestinal permeability [51,52,53]. Moreover, *C. butyricum* is also reported to have an influence on the positive effect of immunomodulation of interleukin-17 (IL-17). An enhanced barrier integrity effect on IL-17 production by γδ T cells (intraepithelial T cells that are one of the components of the first line of defense) in the colonic lamina propria was observed after the implementation of *C. butyricum* [54]. *Clostridium butyricum* was also shown to promote the production of anti-inflammatory lipid metabolites, e.g., palmitoleic acid, prostaglandin metabolites and also pro-resolving mediators in mouse colonic tissues. The mentioned metabolites, such as protectin D1, participate in the promotion of anti-inflammatory T cells secreting IL-10 in the colon. Moreover, microbial components and metabolites are able to stimulate the release of antimicrobial peptides (AMP) and immunoglobulin A (IgA) which could constitute a chemical barrier against microbial pathogens [17,55].

Another positive aspect of the activity of *C. butyricum* comes from reports suggesting their preventive action against inflammation through a mechanism involving type 2 immunity, which may support the treatment of type 1 diabetes. Moreover, literature data indicate an inductive effect of native *Clostridium* spp. such as *C. butyricum* MIYAIRI 588 on regulatory T cells (Tregs) of the colon [38,42]. Jia et al. [56] have suggested that *C. butyricum* CGMCC0313.1 (CB0313.1) could induce pancreatic Tregs cells and consequently suppress diabetes in non-obese diabetic (NOD) mice. Those authors noted that protection could include increased Treg counts, the restoration of Th1/Th2/Th17 cell balance, and a change to a less pro-inflammatory immune environment in the gut, pancreatic lymph node, and pancreas. The increase in α4β7 + (intestinal homing receptor) Treg in the pancreatic lymph node (PLN) suggested that the mechanism may include the increased migration of Treg from the gut to the pancreas. In addition, the sequencing of the 16S rRNA genes showed that CB0313.1 increased the Firmicutes/Bacteroidetes ratio and enriched the Clostridial subgroups and the butyrate-producing bacterial subgroups. These results suggest the beneficial preventive effects on type 1 diabetes and the need for further clinical investigation in this area. 

Tomita et al. [57] noticed that *Clostridium butyricum* therapy may affect the therapeutic efficacy of immune checkpoint inhibitors (ICI). They retrospectively evaluated 118 patients with advanced non-small cell lung cancer treated with immune checkpoint blockage. A survival analysis comparing patients given *C. butyricum* therapy before and after ICI treatment showed the significant prolongation of progression-free survival and overall survival, even in patients who received antibiotic therapy. This study suggests that probiotic therapy with *C. butyricum* may have a positive impact on the therapeutic efficacy of ICI in patients with cancer [57]. Tian et al. [58] investigated the effect of *C. butyricum* on adverse events in lung cancer (LC) patients treated with chemotherapy. Systemic therapy is an aggressive treatment that carries a risk of intestinal damage and gastrointestinal reactions. The authors performed an NGS-based analysis of the intestinal microbiome at baseline and after a three-week treatment with *C. butyricum*, and they found that the incidence of chemotherapy-induced diarrhea was lower in the patient group that was administered *C. butyricum* compared to the placebo group. In addition, Tian et al. [58] found an increase in the amount of bacteria from SCFA-producing genera in the *C. butyricum*-supplemented group, with a concomitant tendency toward decreased pathogenic bacteria which was almost the opposite of the observation in the placebo group. The researchers showed that *C. butyricum* reduced chemotherapy-induced diarrhea in LC patients and decreased systemic inflammatory response [58]. 

Zhou et al. [59] studied the effect of *C. butyricum* on colorectal cancer (CRC) progression. In a study on mice, they demonstrated that the supplementation of *C. butyricum* inhibited the development of CRC in vivo and restrained the proliferation of in vitro CRC cells and the expression of *MyD88* (*MYD88* innate immune signal transduction adaptor) and NF-κB/p65 (nuclear factor-kappa B subunit/nuclear factor NF-kappa-B P65 subunit). Furthermore, the group of mice that had *C. butyricum* administered in vivo showed more apoptotic cells in the tumor tissue, lower levels of IL-6, and higher levels of IL-10 compared to the mice in the control group. The administration of the bacteria altered the composition of the intestinal microflora, which was also enriched in the small intestine and tumor tissue [59]. The inhibition of the CRC cell proliferation cell cycle and the promotion of apoptosis by *C. butyricum* were indicated by Chen et al. [60]. The in vivo inhibition of CRC development was induced by 1,2-dimethylhydrazine dihydrochloride [60]. In addition, the researchers found that the administration of *C. butyicum* reduced inflammation and improved immune homeostasis in induced CRC. A study by Chen et al. [51] additionally indicated that CRC cells showed decreased proliferation and increased levels of apoptosis in the presence of *C. butyricum*. In addition, *C. butyricum* has been shown to inhibit the Wnt/β-catenin signaling pathway (which regulates cellular function such as proliferation, differentiation, migration, genetic stability, apoptosis, and stem cell renewal) and modulate the composition of the gut microbiota [61], and has been associated with a reduction in pathogenic bacteria, as also indicated by Tian et al. [58]. Furthermore, Chen et al. [51] demonstrated that *C. butyricum* increases the number of bile acid biotransforming bacteria and increases the number of beneficial bacteria, including bacteria that produce short-chain fatty acids. In Table 2, the beneficial properties of non-pathogenic *C. butyricum* strains are listed.

## 4. Pathogenic Character of Clostridia and Safety Aspects

Despite the fact that most Clostridia are commensal bacteria, some of them can cause serious health disturbances in humans and animals, such as botulism and necrotic enterocolitis. 

Botulism is a paralyzing disease caused by botulinum neurotoxins (BoNTs). The production of BoNT is generally attributed to *C. botulinum* species [62]. The BoNT molecule is a component of a protoxin complex with several associated protein molecules, all of which are encoded by the botulinum gene cluster. Some of these genes are also found in other *Clostridium* species and some have moved between different plasmids within the same physiological group of Clostridia. This indicates that the horizontal transfer of genes encoding the botulinum cluster occurs between species of *Clostridium*. The abundance of mobile elements is likely connected to accelerated genome plasticity and gene transfer events [63,64,65].

Despite the fact that *C. butyricum* is considered a commensal, non-pathogenic bacterium, cases of botulism in infants have been reported and associated with this species. The first case of botulism in infants caused by *C. butyricum* capable of producing BoNT type E was reported in Italy [62]. The isolated toxigenic organisms differed significantly from *C. botulinum* type E. The visible microorganism in each case resembled *C. butyricum*, but produced a neurotoxin that could not be distinguished from botulinum toxin type E by its effect on mice and its neutralization by botulinum type E antitoxin [15]. Since these cases, *C. butyricum* has been associated with botulism in many other countries, including China, Japan, India, USA, and Ireland [66]. The operon encoding BoNT type E in the toxigenic *C. butyricum* is very similar to the operon encoding BoNT type E in *C. botulinum* group II strains [64].

Aside from botulism, *C. butyricum* strains have been associated with necrotizing enterocolitis (NEC). This was first described by Howard et al. [67] in 1977, who noted *C. butyricum* isolates in stool and blood cultures of preterm neonates. The symptoms of NEC manifest with gastrointestinal bleeding, abdominal distension, mucosal ulcerations, necrosis, portal venous gas, and pneumatosis intestinalis. Generally, NEC causes high morbidity and mortality. The genome sequencing of the pathogenic *C. butyricum* strains identified different toxin genes [38,68]. Cassir et al. [38] identified four genes encoding polypeptides in NEC-associated *C. butyricum* strains that were very similar to hemolysins shared by *Brachyspira hyodysenteriae*, the etiological agent of swine dysentery. Among those hemolysins, the pore-forming β-hemolysin is considered to be the main virulence factor capable of inducing enterocyte necrotic lesions via the culture supernatant. However, the participation of β-hemolysin in the etiology of NEC is not fully elucidated. Studies conducted on an axenic chicken model showed the reproducibility of NEC with *C. butyricum* pathogenic human neonatal-derived strains [69]. The experimental findings suggested that *C. butyricum* could play a primary role in NEC pathogenesis. The bacterium is suspected to ferment carbohydrates depending on the lactase deficiency of preterm neonates [70,71]. The occurrence of NEC caused by *C. butyricum* has been associated mainly with preterm neonates; however, recently, Sato et al. [35] described this in an 84-year-old Japanese man during hospitalization for the treatment of stab wounds.

## 5. Conclusions

*Clostridium* species, especially clusters IV, XIVa, and *C. butyricum*, are commensal bacteria and are common in human and animal intestines. The mentioned groups and *C. butyricum* can exert anti-inflammatory effects and maintain intestinal health by releasing their components and metabolites, especially SCFAs. These microorganisms could find supporting application in IBD, diabetes, and even cancer therapy. Therefore, these strains have broad prospects as probiotics in the future. The next-generation sequencing of 16S RNA genes and metagenomic analyses provide increasing insight into the content of particular clostridial groups in the intestinal biomes of individuals with various diseases. In-depth analyses of the impact of the presence and content of specific groups of Clostridia will certainly contribute to our knowledge of how these bacteria affect disease processes, treatment, and recovery. The properties that Clostridia possess seem to be valuable in the probiotic field. However, despite this, there are still some doubts about the safety of Clostridia application (especially *C. butyricum*) to improve health. Thorough research is still needed on the molecular aspects of physiology and genetic diversity, and on horizontal gene transfer between pathogenic and non-pathogenic strains of Clostridia and related species. 

## Figures and Tables

**Table 2:** Probiotic properties of non-pathogenic *Clostridium butyricum* strains.

	Probiotic Properties	Literature
*C. butyricum*	Production of SCFAs ^1^, especially butyrate; Improving gastrointestinal barrier function; Alleviating inflammation; Preventing Escherichia coli and Clostridioides difficile infections; Preventing the permeation of toxins and antigens; Facilitating the absorption of nutrients, electrolytes and water; Production of anti-inflammatory lipid metabolites (palmitoleic acid prostaglandin metabolites); Stimulation of anti-inflammatory Treg cells; Supporting effect in type 1 diabetes treatment; Positive impact on therapeutic efficacy of ICI ^2^ in patients with cancer; Inhibitory in vivo effect on CRC ^3^ development; Decreasing effect on IL-6 and IL-10 release; Inhibition of Wnt/β-catenin signaling pathway.	[15,17,29,38,39,41,42,44,45,47,48,49,50,51,53,54,56,57,58,59,60]

^1^ SCFA = short-chain fatty acids; ^2^ CI = immune checkpoint inhibitors; ^3^ CRC = colorectal cancer.

## Data Availability

Not applicable.
